# The relationship between weight indices and injuries and mortalities caused by the motor vehicle accidents: a systematic review and meta-analysis

**DOI:** 10.5249/jivr.v12i1.1198

**Published:** 2020-01

**Authors:** Homaie Rad Enayatollah, Naema Khodadady-Hasankiadeh, Leila Kouchakinejad-Eramsadati, Fatemeh Javadi, Zahra Haghdoost, Marieh Hosseinpour, Maryam Tavakoli, Ali Davoudi-Kiakalayeh, Zahra Mohtasham-Amiri, Shahrokh Yousefzadeh-Chabok

**Affiliations:** ^*a*^ Social Determinants of Health Research Center, Guilan University of Medical Sciences, Rasht, Iran.; ^*b*^ Guilan Road Trauma Research Center, Guilan University of Medical Sciences, Rasht, Iran.; ^*c*^ Department of Epidemiology and Public Health, Swiss Tropical and Public Health Institute, Basel, Switzerland.

**Keywords:** Body Mass Index Overweight, Obesity, Injuries, Mortality, Motor vehicle accidents

## Abstract

**Background:**

The relationship between weight indices and injuries and mortality in motor vehicle accidents is unknown. Systematic review studies addressing the collection and analysis of the relationship in investigations are very limited. The purpose of this systematic review is to deter-mine the relationship between BMI, obesity and overweight with mortality and injuries and their severity and vulnerable organs after the motor vehicle accident.

**Methods:**

The databases (MEDLINE/PUBMED, EMBASE, Web of Science, etc) were searched for relevant abstracts using certain keywords. Of all the articles, similar ones were removed considering different filters. The collected data were entered into the STATA SE v 13.1. The heterogeneity of the data was analyzed using i2 statistics. In addition, the estimates of the study were done based on the age group (children and adults) and the impact of obesity on different regions of the body.

**Results:**

A direct relationship was observed between the overall BMI and the degrees of injuries (CI=0.503-1.139), and mortality due to motor vehicle accident (CI=1.267-1.471). A positive relationship was found between obesity and AIS+2 (CI=0.653-1.426), and AIS+3 (CI=1.184-1.741), and ISS (CI=1.086-1.589). Also, a negative relationship between overweight and inju-ries rates, and a direct relationship between overweight and mortality (CI=0.979-1.167), and injuries with index of AIS+2 (CI=1.178-0.768) and AIS+3 (CI=0.48-2.186) were found.

**Conclusions:**

The prediction of injury, mortality and severity of injuries in the motor vehicle accident by the variable of obesity and overweight determines the need to design prevention programs for this vulnerable group at all levels.

## Introduction

Both motor vehicle accident (MVA) and obesity are among the most important causes of mortality and morbidity in the modern world. MVA is a general problem that affects all countries in the world, especially developing countries. According to the World Health Organization (WHO), about 1.25 million people died due to MVA in 2013. Although mortality rate caused by MVA is slowly declining worldwide, this trend in many developing countries is rising.^1,2^

In 2014, of nearly 1.2 billion overweight adults all over the world, 600 million people were obese. The World Health Organization has announced that obesity has doubled in the world over the past three decades, and is still growing in the world.^3,4^ Obesity and overweight are among the major public health problems globally.^1^ It is a complex and serious condition with social and psychological dimensions that involves all ages and socio-economic groups.^5^

The most commonly used method for measuring overweight and obesity is the Body Mass Index (BMI). The BMI is obtained by dividing the weight (in kilograms) by a square of height (in meters). It is the most useful measure for overweight and obesity because it is used in the both sexes in all ages equally.^6^ The proposed standard method of the World Health Organization has defined the BMI as equal to or more than 30 as obesity, and the BMI over 25 as overweight.^7,8^ In children; however, percentiles are used to estimate overweight. BMI between 85th and 95th percentiles are defined as overweight, above 95th percentile for age and sex as obesity.^9^

Many illnesses develop with obesity that each constitute a major threat to the individual's life. These diseases include heart disease, diabetes, osteoarthritis, and various types of cancer and depression. They can affect consequences of traumas and MVA.^1,3^

The effects of obesity on the outcomes and mortality have much been discussed.^2,4^ A meta-analysis entitled “Consequences of Obesity in Traumatic Patients” showed that the consequences of obesity in spinal column trauma, the prolonged duration of surgery, increased blood loss, increased infection site, and neurological damages had significant differences in obese and non-obese individuals. However, no significant difference was found between the two groups in the mortality rate and deep-vein thrombosis. In this study, MVA-induced traumas were not separated.^3^

Various reports have been published on the effects of obesity on the outcomes of the traumas, in which contradictory results have been observed. For example, the results of the study showed that no difference in mortality was found between obese and non-obese people.^5^ In another study, mortality rate in obese patients with penetrating trauma was significantly higher than that in patients with normal weight.^6^ On the other hand, obesity may be supportive and reduce mortality due to hitting. For example, Diaz et al., showed that a BMI over 40 has not been associated with the deaths of patients in intensive care units.^7^ Also, evidence suggests that obese patients are significantly more at risk for cardiovascular and respiratory problems following traumas.^8^

A study reported that an inverse statistically significant relationship between obesity and agility, and also between obesity and speed, which means that by increasing BMI, the agility and speed of the person's activity decreases.^9^

Additionally, obese people are reported to use seat belts less frequently because of their discomfort, which can lead to more severe injuries when driving accidents.^10^

However, a study was conducted on the relationship between obesity and driving accidents in 2014 to answer the question of whether obesity increases the risk of injuries and deaths caused by motor vehicle accidents.^1^ Due to the oldness of this study, increased number of printed articles in this period and, on the other hand, a small number of review papers in this review (9 papers), the study needs to be repeated. The study should take into account all age groups and define different criteria for differentiating obesity in children from that in adults. Therefore, the purpose of this study was to determine the relationship between BMI, obesity and overweight with mortality and the degree of injuries and their severity and vulnerable organs following MVA.

## Materials and Methods

**Figure 1:**
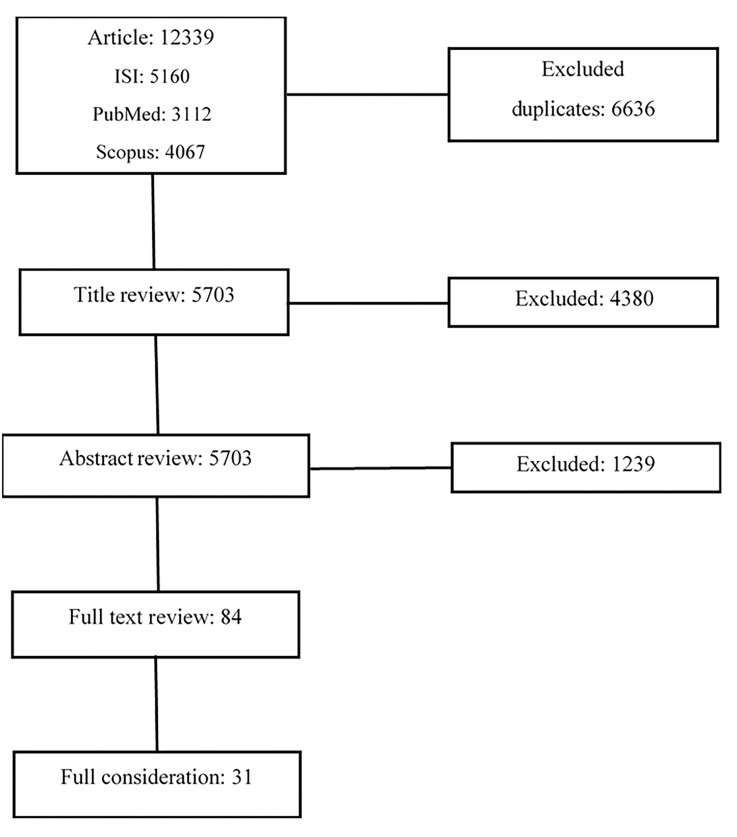
Reviewing studies on the impact of overall BMI on Injuries.

First, the status of published review articles regarding the relationship between obesity and severity of driving accidents was examined. After similar studies were confirmed not to be conducted or not to be old, the exact strategy of searching in databases was defined based on these keywords. (Figure 1)

The Inclusion criteria for the studies were as follows:

- MVA-related studies published in an English-language journal after 2000;

- Existing at least one of the health outcome associated with injuries, (ISS or AIS) or mortality, in pedestrians, cyclists, motorcyclists, or car drivers and its passengers;

- Existing at least one index related to obesity and overweight in studies;

- Using a regression model or using statistics on the relationship between obesity and severity of accidents;

The relationship between the obesity measurement unit and accidents as OR, RR, HR;

- Existing enough data in the study to convert other relationships.

To conduct this study, databases of SCOPUS, Web of Science, and PUBMED, were searched on July 12, 2017. The search strategy of SCOPUS data base is shown in the Appendix 1

**Appendix 1:**
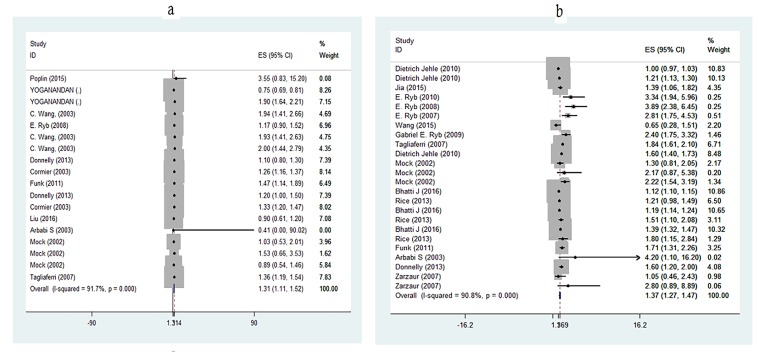

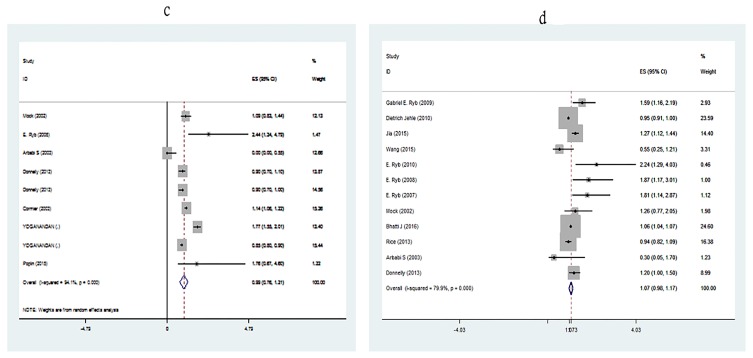

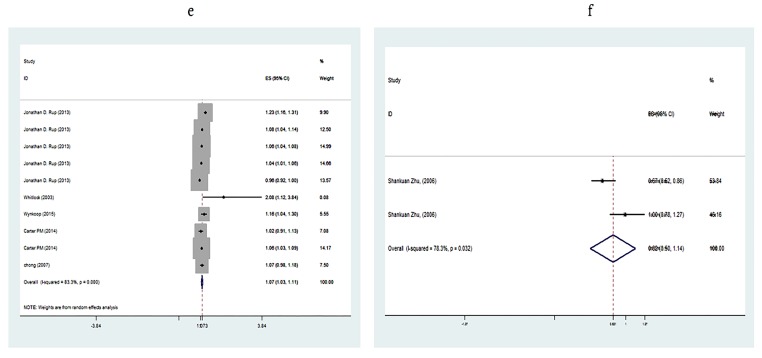
( TITLE-ABS-KEY ( ( motor* OR vehicl* OR pedestrian* OR car* OR incident* OR bicycl ) ) ) AND ( TITLE-ABS-KEY ( ( fatalit* OR deat* OR dead* OR victim* OR deceas* OR ascot OR iss OR trauma* OR gcs OR los OR ( "length of stay" ) OR ( "Glasgow coma scale" ) OR ( "injury severity score" ) ) ) ) AND ( ( TITLE-ABS-KEY ( obesit* OR adiposit* OR flesh* OR overweight* OR plumpness* OR heavy OR fat* OR rotundity* OR bmi ) ) ) AND ( TITLE-ABS-KEY ( crash OR accident OR coincidence OR hit ) ) AND ( ( TITLE-ABS-KEY ( road* OR traffic* OR way* OR path* OR street* OR route* OR avenue* OR track* ) ) ) AND ( LIMIT-TO ( DOCTYPE , "ar " ) ) AND ( LIMIT-TO ( PUBYEAR , 2017 ) OR LIMIT-TO ( PUBYEAR , 2016 ) OR LIMIT-TO ( PUBYEAR , 2015 ) OR LIMIT-TO ( PUBYEAR , 2014 ) OR LIMIT-TO ( PUBYEAR , 2013 ) OR LIMIT-TO ( PUBYEAR , 2012 ) OR LIMIT-TO ( PUBYEAR , 2011 ) OR LIMIT-TO ( PUBYEAR , 2010 ) OR LIMIT-TO ( PUBYEAR , 2009 ) OR LIMIT-TO ( PUBYEAR , 2008 ) OR LIMIT-TO ( PUBYEAR , 2007 ) OR LIMIT-TO ( PUBYEAR , 2006 ) OR LIMIT-TO ( PUBYEAR , 2005 ) OR LIMIT-TO ( PUBYEAR , 2004 ) OR LIMIT-TO ( PUBYEAR , 2003 ) OR LIMIT-TO ( PUBYEAR , 2002 ) OR LIMIT-TO ( PUBYEAR , 2001 ) OR LIMIT-TO ( PUBYEAR , 2000 ) ) AND ( LIMIT-TO ( LANGUAGE , "English " ) ) Appendix two: Forest plots of the estimated effect sizes. a. Relationship between Obesity and Total Body Injuries b. Relationship between Obesity and Mortality c. The Relationship between Overweight and Total Body Injuries d. The Relationship between overweight and morality e. The relationship between BMI and injury f. The relationship between BMI and mortality

Then, of the total of three databases, 12339 articles (ISI: 5160 articles, Scopus: 4067 articles, and Pubmed: 3112 articles) were obtained. After removing similar articles, 6636 articles left. The repetitive articles was removed based on both the title and the authors of the articles. At this point, by sorting out the names of the authors of the articles, those using a same database or the data of a same survey study were deleted.

Subsequently, the three researchers removed studies that clearly had titles unrelated to trauma and MVA, leaving 1323 articles for the abstract review. Five researchers (in two groups of two and one reviewer) reviewed abstract of articles, and unrelated studies were deleted. Then, disputed articles were resolved by the reviewer and the two groups in a session (with a difference of 9.6%). Finally, 84 articles remained in order to examine the full texts. Subsequently, they were downloaded from the databases. The four researchers examined the articles and removed the unrelated articles. At the end of this step, the articles were numbered. After reviewing the full texts of the articles, and deleting the articles that did not have inclusion criteria, at last 31 articles were determined for collecting the necessary data and information.^11-41^

An Excel-based checklist was prepared for gathering information, and the required variables were collected. Then, all required information were separated from various studies, such as output data of OR, HR, RR from patterns estimation related to age, gender, degree of education, type of disease, etc., related coefficients, significance, study sample size, the units of measurement, etc.

The collected data were entered into the STATA SE v 13.1 software for meta-analysis. The heterogeneity of the data was analyzed using i2 statistics. Based on this statistic, the estimated type was selected with constant or random effects. Also, the results of various statistics related to the lack of studies bias, etc. were examined. In addition, the estimates of the study were done based on the age group (children and adults) and the impact of obesity on different regions of the body.

## Results

Table 1 shows the characteristics of systematic review studies. 28 out of 31 systematic reviews were conducted in the United States, and three studies in Taiwan, Sweden and New Zealand. The total sample size was 24022527, which is considered statistically desirable. The US NASS-CDS database was used by 12 studies, and the CIREN database was used by eight studies. Of 31 studies, 14 were published after 2010, and the rest before 2010. Three studies were conducted on children and adolescents, and seven studies in all age groups, and the remaining in adults. Also, in seven out of the 31 studies, no reports of adjustment of confounding variables were observed, but age, gender, fastening seat belts, seats used by passengers, type of accident, type of vehicle, etc. were included as control variables in other studies. All of them were considered in the retrospective cohort study.

**Table 1:** Characteristics of the studies entered into the Systematic Review.

author	pub-year	country	design	population	sample size	type of crash	data source	adjustments
Arbabi S	2003	USA	retrospective cohort study	age +15	189	Motor vehicle	Michigan data	age, sex
Carter PM	2014	USA	retrospective cohort study	age +17	18371	all types	NASS-CDS dataset	(age, BMI, gender),
Chong	2007	USA	retrospective cohort study	no age limited	137	Motor vehicle	CIREN U of M	gender, age, height, weight
Cormier	2003	USA	retrospective cohort study	age +18	28096	whole	NASS-CDS	no adjustment
Donnelly	2013	USA	retrospective cohort study		10303270	overall	NASS CDS and CIREN	age, sex, seat belt use, seat position, vehicle crush, impact type, and intrusion
Funk	2011	USA	retrospective cohort study	age+16	2496298	rollover crashes	NASS-CDS	age, type of vehicle, sex, seat belt…
Haricharan	2009	USA	retrospective cohort study	2-5 years	9 million	motor vehicle	NASS/CDS	
Liu	2016	Taiwan	case control	20–65 years old	3167	motorcycle	Trauma Registry System	not determined
Jia	2015	Sweden	cohort retrospective		743398	motor vehicle	national register	intelligence quotient, systolic blood pressure, socioeconomic position, Muscle strength
Mock	2002	USA	retrospective cohort study	15 years and older	26727	tow-away crashes of cars, light trucks, vans and sport utility vehicles.	NASS-CDS	occupant age, gender, seatbelt use, occupant seat position, and vehicle curb weight
Pollack	2008	USA	retrospective cohort study	9-15 years old	3232	motor vehicle	Partners for Child Passenger Safety (PCPS) study	age in years, gender, restraint type, seating position, passenger airbag exposure, vehicle type and model year, direction of initial impact, crash severity, rollover, and driver airbag deployment
Poplin	2015	US	retrospective cohort study	age+16	25407	car	NASS-CDS	occupant age
E. Ryb	2010	US	retrospective cohort study	AGE+15	1226	car+truk+suv+van	CIREN	other occupant and crash factors
E. Ryb	2008	US	retrospective cohort study	age+16	1615	car	CIREN	patient and crash factors
E. Ryb	2007	US	retrospective cohort study	16-81	1261	car	CIREN	age + gender+ BMI
C. Wang	2003	US	retrospective cohort study	19-65	67	motor vehicle	CIREN	belt use وcrash severity ,age
Wang	2015	US	retrospective cohort study	AGE+19	14453	motor vehicle	CDS	age, sex, race, family income, education attainment, alcohol drinking, cigarette smoking, marital status and self-evaluated health
Whitlock	2003	New Zealand	retrospective cohort study	16 to 88	139	motor vehicle	national health databases	Age, sex, alcohol intake, and driving exposure, area of residence, driving exposure, marital status and occupational status,
Wynkoop	2015	US	retrospective cohort study	NO REPORT/AGE VEICLE	10000	motor vehicle	NASS-CDS	no report
YOGANANDAN	2014	US	retrospective cohort study	NO REPORT/AGE VEICLE	519195	CAR+TRUK+SUV+VAN	NASS-CDS	Occupant, vehicle, and crash-related factors.
Zarzaur	2007	USA	retrospective cohort study	16 years and older	9313	occupants	NASS-CDS	1) change in velocity; 2) rollover crash; 3) other vehicle class
Pavan P. Zaveria,	2009	USA	retrospective cohort study	age 2–17 years	335	motor vehicle	(CIREN)	
Shankuan Zhu,	2006	USA	retrospective cohort study	age+16	22107	motor vehicle	NASS	Age, BMI, seat belt use, airbag deployment, manner and type of collision, alcohol use, drug use, vehicle age and weight, and road speed limit. T
Bhatti J	2016	USA	retrospective cohort study	age +15	534887	cars	FARS	age and gender
Bansal,	2009	USA	retrospective cohort study	at least 13 years old	424	motor vehicle	CIREN	
Reiff	2004	USA	retrospective cohort study	age+18	15237	all types	SUDAAN	gender, age, restraint use, and seating position
Rice	2013	USA	retrospective cohort study	age+16	41296	drivers	FARS and VINDICATOR	status and vehicle type sex, seat belt use, head-on collision
Jonathan D. Rup	2013	USA	retrospective cohort study	age+16	36290	drivers	NASS-CDS	frontal model
Gabriel E. Ryb	2009	USA	retrospective cohort study	age+16	1888	drivers	CIREN	
Tagliaferri	2007	USA	retrospective cohort study		5918	drivers	NASS	sex, age, seat belt use
Dietrich Jehle	2010	USA	retrospective cohort study		158584	drivers	FARS	age, lighting, weather, and the presence of air bag deployment car type, restraint use, alcohol and drug use,

**1. Relationship between BMI and obesity with injuries and mortality**

1.1. Relationship between overall BMI and the occurrence of injuries (morbidity)

The studies on the relationship between overall BMI and injuries found that overall BMI has a positive relationship with the incidence of injuries in MVA (Table 2).

**Table 2:** Relationship between overall BMI and injuries.

Author	effect size	95% lower limit	95% upper limit	weight
Jonathan D. Rup (2013)	1.234	1.162	1.31	9.9
Jonathan D. Rup (2013)	1.083	1.041	1.139	12.5
Jonathan D. Rup (2013)	1.062	1.041	1.083	14.99
Jonathan D. Rup (2013)	1.041	1.01	1.062	14.66
Jonathan D. Rup (2013)	0.961	0.923	1	13.57
Whitlock (2003)	2.08	1.12	3.84	0.08
Wynkoop (2015)	1.162	1.039	1.3	5.55
Carter PM (2014)	1.015	0.914	1.127	7.08
Carter PM (2014)	1.063	1.03	1.094	14.17
chong (2007)	1.073	0.976	1.178	7.5
Pooled effect size	1.069	1.030	1.107	100

Heterogeneity χ2 = 53.81 , p = 0.000

1.2. Relationship between overall BMI and mortality

Overall BMI has also an inverse relationship with mortality due to MVA (Table 3).

**Table 3:** The Relationship between overall BMI and mortality.

Author	effect size	95% lower limit	95% upper limit	weight
Shankuan Zhu (2006)	0.671	0.523	0.861	53.84
Shankuan Zhu (2006)	0.997	0.781	1.271	46.16
effect sizes	0.821	0.503	1.139	100

Heterogeneity χ2 = 4.61 , p = 0.032

1.3. Relationship of obesity and injuries (Obesity-overall injury)

The relationship between obesity and injuries has been expressed differently, and studies reporting a negative relationship between obesity and injuries are more common. The index of severity of injuries in these studies includes AIS and ISS. The relationship between obesity and severity of injuries was reported to be negative in four studies (Mock, Grossman et al., 2002, Arbabi, Wahl et al., 2003, Yoganandan, Arun et al., 2014, Liu, Rau et al., 2016). In other studies, this relationship was positive. Also, the pooled effect coefficient is 1.314, which indicates a positive and strong relationship between obesity and total body injuries (Table 4).

**Table 4:** Relationship between obesity and total body injuries.

Author	effect size	95% lower limit	95% upper limit	weight
Poplin	3.55	0.83	15.2	0.08
Yoganandan	0.75	0.69	0.81	8.26
Yoganandan	1.9	1.64	2.21	7.15
C. Wang	1.939	1.415	2.656	4.69
E. Ryb	1.17	0.899	1.522	6.96
C. Wang	1.927	1.411	2.632	4.75
C. Wang	2.004	1.44	2.788	4.35
Donnelly	1.1	0.8	1.3	7.39
Cormier	1.26	1.16	1.37	8.14
Funk	1.47	1.14	1.89	6.49
Donnelly	1.2	1	1.5	7.39
Cormier	1.33	1.2	1.47	8.02
Liu	0.9	0.61	1.2	7.08
Arbabi S	0.407	0.002	90.017	0
Mock	1.03	0.53	2.01	3.96
Mock	1.53	0.66	3.53	1.62
Mock	0.89	0.54	1.46	5.84
Tagliaferri	1.36	1.19	1.54	7.83
Effect size	1.314	1.111	1.518	100

1.4. Relationship between obesity and mortality 

The positive relationship between obesity and mortality was reported in all studies, except in two studies (Jehle, Gemme et al., 2012, Wang, Obi et al. 2015). The pooled effect coefficient was 1.369 showing that obesity has a direct relationship with mortality due to MVA (Table 5).

**Table 5:** Relationship between obesity and mortality.

Author	effect size	95% lower limit	95% upper limit	weight
Dietrich Jehle (2010)	0.996	0.966	1.026	10.83
Dietrich Jehle (2010)	1.212	1.128	1.302	10.13
Jia (2015)	1.39	1.06	1.82	4.35
E. Ryb (2010)	3.34	1.94	5.96	0.25
E. Ryb (2008)	3.89	2.38	6.45	0.25
E. Ryb (2007)	2.81	1.75	4.53	0.51
Wang (2015)	0.65	0.28	1.51	2.2
Gabriel E. Ryb (2009)	2.4	1.75	3.32	1.46
Tagliaferri (2007)	1.84	1.61	2.1	6.71
Dietrich Jehle (2010)	1.599	1.402	1.734	8.48
Mock (2002)	1.3	0.81	2.05	2.17
Mock (2002)	2.17	0.87	5.38	0.2
Mock (2002)	2.22	1.54	3.19	1.34
Bhatti J (2016)	1.12	1.1	1.15	10.86
Rice (2013)	1.21	0.98	1.49	6.5
Bhatti J (2016)	1.19	1.14	1.24	10.65
Rice (2013)	1.51	1.1	2.08	3.11
Bhatti J (2016)	1.39	1.32	1.47	10.32
Rice (2013)	1.8	1.15	2.84	1.29
Funk (2011)	1.71	1.31	2.26	3.25
Arbabi S (2003)	4.2	1.1	16.2	0.02
Donnelly (2013)	1.6	1.2	2	4.08
Zarzaur (2007)	1.05	0.46	2.43	0.98
Zarzaur (2007)	2.8	0.89	8.89	0.06
Pooled	1.369	1.267	1.471	100

Heterogeneity χ2 = 250.45 p = 0.000

**2. Relationship between obesity and AIS higher than 2 and 3 and ISS**

2.1. Relationship between obesity and AIS more than 2 (AIS+2)

 In four studies that examined the relationship between obesity and AIS higher than 2, an inverse relationship was reported in a study, and was positive in other studies. In general, a positive relationship between obesity and AIS more than two was observed (pooled results coefficient=1.04) (Table 6).

**Table 6:** The relationship between obesity and AIS higher than 2.

Author	effect size	95% lower limit	95% upper limit	weight
Poplin	3.55	0.83	15.2	0.29
YOGANANDAN	0.75	0.69	0.81	34.84
Donnelly	1.1	0.8	1.3	30.63
Cormier	1.26	1.16	1.37	34.24
pooled results	1.04	0.653	1.426	100

Heterogeneity χ2 = 71.82 p = 0.000

2.2. Relationship between obesity and AIS higher than 3 (AIS+3)

When AIS was more than 3, the relationships were more homogeneous, so that the results of all four studies indicated a direct relationship between obesity and AIS higher than 3. Also, the pooled coefficient was 1.463 (Table 7).

**Table 7:** The relationship between obesity and AIS higher than 3 (AIS+3).

Author	effect size	95% lower limit	95% upper limit	weight
Yoganandan	1.9	1.64	2.21	24.1
Funk	1.47	1.14	1.89	20.33
Donnelly	1.2	1	1.5	25.59
Cormier	1.33	1.2	1.47	29.98
pooled effect	1.463	1.184	1.741	100

Heterogeneity χ2 = 15.44 p = 0.001

2.3. Relationship between obesity and ISS

Of the ten studies, an inverse relationship between obesity and ISS was observed in three studies, a positive and significant relationship was found in other studies (Mock, Grossman et al., 2002, Arbabi, Wahl et al., 2003, Liu, Rau et al 2016). In addition, the pooled effect size was 1.338, which indicates that the severity of injuries in obese individuals is higher than that in other people (Table 8).

**Table 8:** The relationship between obesity and ISS.

Author	effect size	95% lower limit	95% upper limit	weight
Liu	0.9	0.61	1.2	16.16
C. Wang,	1.939	1.415	2.656	9.17
E. Ryb	1.17	0.899	1.522	15.76
Arbabi S	0.407	0.002	90.017	0
C. Wang,	1.927	1.411	2.632	9.33
C. Wang,	2.004	1.44	2.788	8.33
Tagliaferri	1.36	1.19	1.54	18.88
Mock	1.03	0.53	2.01	7.42
Mock	1.53	0.66	3.53	2.68
Mock	0.89	0.54	1.46	12.26
Pooled results	1.338	1.086	1.589	100

Heterogeneity χ2 = 24.03 p = 0.004

**3. Overweight and injuries and deaths**

3.1. Overweight-injury 

The relationship between overweight and total body injuries showed that the effect sizes were more inconsistent than the relationship between obesity and injuries. Of a total of 9 coefficients, 4 coefficients reported a negative r elationship between overweight and injuries (Arbabi, Wahl et al. 2003, Donnelly, Griffin et al. 2014, Yoganandan, Arun et al. 2014) ( Table 9).

**Table 9:** Relationship between overweight and total body injuries.

Author	effect size	95% lower limit	95% upper limit	weight
Mock	1.09	0.83	1.44	12.13
E. Ryb	2.44	1.241	4.792	1.47
Arbabi S	0.004	0	0.549	12.66
Donnelly	0.9	0.7	1.1	13.87
Donnelly	0.9	0.7	1	14.56
Cormier	1.14	1.06	1.22	15.26
YOGANANDAN	1.77	1.55	2.01	13.4
YOGANANDAN	0.85	0.8	0.9	15.44
Poplin	1.76	0.67	4.6	1.22
effect size	0.988	0.762	1.214	100

Heterogeneity χ2 = 135.92 , p = 0.000

3.2. Relationship between overweight and mortality as compared to obesity

The number of non-homogeneous and contradictory studies on the relationship between overweight and mortality was higher. Of the 13 studies, 5 reported an inverse relationship between overweight and mortality (Arbabi, Wahl et al., 2003, Jehle, Gemme et al., 2012, Rice and Zhu 2014, Wang, Obi et al. 2015). Also, pooled effect size was 1.073 in these studies, which indicates a direct relationship between overweight and mortality due to MVA (Table 10).

**Table 10:** The relationship between overweight and mortality.

Author	effect size	95% lower limit	95% upper limit	weight
Gabriel E. Ryb	1.59	1.16	2.19	2.93
Dietrich Jehle	0.952	0.911	0.995	23.59
Jia	1.27	1.12	1.44	14.4
Wang	0.55	0.25	1.21	3.31
E. Ryb	2.24	1.29	4.03	0.46
E. Ryb	1.87	1.17	3.01	1
E. Ryb	1.81	1.14	2.87	1.12
Mock	1.26	0.77	2.05	1.98
Bhatti J	1.06	1.04	1.07	24.6
Rice	0.94	0.82	1.09	16.38
Arbabi S	0.3	0.05	1.7	1.23
Donnelly	1.2	1	1.5	8.99
Pooled results	1.073	0.979	1.167	100

Heterogeneity χ2 = 54.70 , p = 0.000

**4. Relationship between overweight and AIS over 2 and 3 and ISS**

4.1. The relationship between overweight and AIS data higher than 2 (AIS+2)

 Of the four data entered in this section, two coefficients reported inverse relationship between overweight and AIS higher than two. The pooled effect coefficient was reported 0.973, which means that the probability of injury with an AIS index more than two in overweight individuals is higher (Table 11).

**Table 11:** The relationship between overweight and injuries for AIS data higher than 2 (AIS+2).

Author	effect size	95% lower limit	95% upper limit	weight
Poplin	1.76	0.67	4.6	1.06
Donnely	0.85	0.8	0.9	35.02
Yoganandan	0.9	0.7	1	30.01
Cormier	1.14	1.06	1.22	33.91
pooled results	0.973	0.768	1.178	100

Heterogeneity χ2 = 67.74 , p = 0.000

4.2. Relationship between overweight and injuries for AIS data higher than 3(AIS+3)

The relationship between overweight and injuries for AIS more than 3 shows a positive relationship. This means that the likelihood of accidents with AIS more than 3 is higher in overweight people (Table 12).

**Table 12:** The relationship of overweight and injuries for AIS data higher than 3.

Author	effect size	95% lower limit	95% upper limit	weight
Yoganandan	1.77	1.55	2.01	49.78
Donnelly	0.9	0.7	1.1	50.22
pooled results	1.333	0.48	2.186	100

Heterogeneity χ2= 31.30 p = 0.000

4.3. Relationship between overweight and injuries for the ISS Index

 The relationship was investigated in three studies. The results of the studies showed that this relationship is direct in two studies and indirect in one study. The findings of the pooled effect also showed a negative relationship 891 between overweight and severity of injuries with the coefficient of 0.891 (Table 13).

**Table 13:** The relationship between overweight and injuries for the ISS Index.

Author	effect size	95% lower limit	95% upper limit	weight
E. Ryb	2.44	1.241	4.792	18.24
Arbabi S	0.004	0		41.03
Mock	1.09	0.83	1.44	40.74
pooled results	0.891	-0.114	1.896	100

Heterogeneity χ2 = 31.49 , p = 0.000

**5. Relationship between BMI, obesity, and overweight with damage to the body organs**

A relationship was observed between overall BMI and external organ injuries (coefficient=1.062). The findings of the study also showed that overweight had an inverse relationship with internal organ injuries (coefficient=0.7). Obesity also had an inverse relationship with the injuries of these organs (coefficient=0.992). However, the effect of overweight on injuries of internal organs was more than that of obesity. The results of the study on the external organs were opposite. Overweight had a direct relationship with the severity of external organ injuries with a coefficient of 1.042, while the effect of obesity on them was much higher with a coefficient of 1.399.

**Heterogeneity and results of meta-regression:**

As shown in the tables, the results of meta-analysis showed that there were heterogeneity between studies in all of the estimated models. The χ2 statistics of heterogeneity was significant in all estimated pooled effect sizes and confirmed the presence of heterogeneity. To find the reason of heterogeneity, meta regression models were estimated. In these models, the dependent variable was the effect sizes and independent variables contained “percentage of females to total sample size in each article”, “average of age in each article” and “being adjusted by seat belt use or not”. The results of meta regression models showed that only in one pooled effect size the results were related to average of age (Relationship between Overweight and Mortality) (coefficient= 0.129, P-value=0.012) and in one model, significant relationship was found between effect sizes and percentage of females (Relationship between Obesity and ISS) (coefficient=0.058, P-value=0.033). No differences were found in the effect sizes in studies which adjusted seat belt use and other studies. 

## Discussion

Only one systematic review study has been conducted with similar title during the last five years, ^1^ in which the number of included articles was much lower than the recent one. Despite the inconsistency of the articles, this study was able to answer the main hypotheses of the goal studies.

In this study, body injuries were associated with overall BMI. Since obesity has been associated with direct and severe injuries, and overall BMI is a misleading index because of involving a wide range of thin to obese, the relationship between obesity and general injuries is addressed. In a study with a paradoxical result, obesity reduced the number of injuries and mortality.^42^ In a similar study, the probability of occupational trauma was higher in obese people, and heavyweight individuals were referred to clinics more than other people for the treatment of occupational trauma.^43^ It can be argued that visual impairment, hyperglycemia, hyperlipidemia, and hypertension,^44^ cardiovascular diseases,^45^ apnea,^46^ and the possibility of poor general health^47^ can be seen in obese individuals (with higher BMI). The comorbidity of these diseases with obesity can lead to aggravate cardiovascular, respiratory and apnea signs and symptoms. These patients are more likely to be transported to emergency wards compared to normal-weight patients, and registered and reported as an injured patient, and therefore the casualties rise. Meanwhile, a recent study has shown that reporting violent driving offenses by obese people to the police department is more common than that by non-obese individuals,^47^ that is, it is likely to be obesity as a causative agent of crime and driving violations such as not wearing a seat belt, and it may have a close relationship with high occurrence of injuries in these people. Therefore, maintaining public health and a normal weight can reduce the incidence of injuries caused by MVA.

On the other hand, BMI had an inverse relationship with the mortality rate due to MVA. Because of the weakness of this index and its variability, depending on the number of thin or low weight people entering the study, and considering the direct relationship between obesity and mortality due to the MVA, we will discuss this latter case. The evidence of a meta-analysis strongly supports the relationship between obesity with poor prognosis and motility in trauma-stricken people.^3^ Formerly, obesity was known to increase the risk of death after the traumatic brain injury (Obesity class II and III), and BMI>35 was an independent predictor of TBI-induced hospital mortality.^48,49^ Also, obese drivers are more subject to MVA-induced mortality, regardless of using safety equipment.^50^ In another study, hospital mortality was higher in high-BMI traumatic patients because thrombotic complications increased due to less mobility caused by obesity and decreased participation in physiotherapy in these patients.^51^ Also, a risk of pneumonia and acute respiratory distress syndrome increased,^52^ which indicates a poor prognosis in this group.

The present study also showed a positive relationship between obesity and AIS higher than two and three, and the ISS index. It means that obesity increases the severity of injuries in the MVA, since obese people wear seat belt less frequently according to a study,^53^ as well as the highest incidence of seat belt disproportion has been reported in these individuals.^54^ These two issues make them more susceptible to more serious injuries.^55^ The automotive industry must provide the fully intelligent safety equipment for passengers in all weight groups.^56^ The obese people have increased risk in airway management in a pre-hospital setting due to the anatomical change of the airway, neck shortness, limitation in cervical extension, and fatty layer in the pharynx wall. It is also difficult to maintain the airways of these people for surgery because of changes in the pulmonary mechanism and circulatory system.^57^ It has been suggested that BMI> 35 (obesity) be considered as a precursor variable, and should be continuously measured and evaluated.

A direct relationship was observed between overweight and mortality due to MVA. In a study, however, people with overweight and obesity showed lower mortality significantly compared to the people with normal weight one year after admission due to cardiac failure, myocardial infarction (MI), and pneumonia. However, no accurate information was available on the immediate mortality of these individuals.^58^ Another study with consistent findings indicated that high weight is an independent predictor of cardiovascular and respiratory problems following trauma. Overweight is a risk factor for cardiac arrest, acute respiratory distress syndrome, pulmonary embolism, deep vein thrombosis, and unplanned intubation.^8^ These are the problems that increase the risk of death in these people. Transportation of heavy-weight injured individuals is a challenge for the EMS because there are typically few staff members to transport them to the ambulance and then to the hospital. These kinds of patients are transported by robots in developed countries to face this challenge.^59^

In the present study, there was a negative relationship between overweight and injury. A positive relationship was found between overweight and injuries with AIS index higher than two and three, respectively and a negative relationship between overweight and ISS. In the case of the ISS, no evidence confirming our finding was not found. However, in the case of a negative relationship between overweight and injury degrees, it seems that overweight does not increase the likelihood of injury, but it affects the severity of these injuries. In a study, those with higher BMI significantly suffered from injuries with AIS index more than 3 in the lower extremities and Thorax.^60^ Given that studies that specifically examine the effect of overweight on injury were not found, we used the studies on obese people to interpret. Trauma-stricken obese people need for postoperative care, basic equipment, total length of stay, days mechanical ventilation, ICU stay days, rehabilitation days, and more caregivers.^8,48,49^

Overweight has an inverse relationship with internal organ injuries and a direct relationship with external organ injuries. The same relationship was observed in obesity, but the effect of obesity on the severity of external organs injuries was higher than that of overweight. In a study, however, obesity is a known risk factor for severe abdominal injuries, especially liver injury. These types of injuries increase the length of hospitalization in the intensive care unit and the overall length of hospitalization. This difference in outcomes may be due to the possibility that steatosis or fatty liver in obese people is likely to prevail and can increase the outcomes of injury in these individuals.^61^ Studies on simulators also confirmed that those with a higher weight are more likely to be injured in the extremities and chest. However, the severity of the injury in the extremities is due to the disproportion of the seat belt. The presence of adipose tissue in the abdomen of the obese persons displaces seat belt from its normal position and increases the severity of the injury.^55^ A study with consistent results showed that the compound fractures of the radius bone were higher with any increase in BMI. In spite of this fact, these individuals experienced less inability than normal weight persons.^62^

In the present study, overall BMI also had a direct relationship with external organ injuries, but the studies dividing BMI had some limitations. Therefore, we cannot properly interpret this finding. However, analyzing data from a database including 140817 children data showed that high BMI was associated with severe injuries at the extremities, and milder injuries in the head, abdomen, and chest and spine.^63^ Another study showed that the damage to rear obese passengers who did not wear the seat belt was more severe than those with the same weight in front passengers of the vehicle.^64^ These results also emphasize that more attention should be paid to wear the seat belt in obese people. It is recommended to use a modern seat belt system with shoulder and waist restraint that can effectively reduce body rotation.^55^

Overall BMI and obesity were reported separately, that was the main limitation of the present study. The next constraint was to consider mortality in general and not to separate pre-hospital from the hospital deaths. It was also not possible to separate the relationship between obesity and overweight with mortality and injuries in pedestrians, drivers, and passengers, etc. Furthermore, we add English articles in the systematic review which might be resulted to bias. Additionally, the data of those who wore or did not wear seat belt were not addressed separately.

## Conclusion

Obesity and overweight directly increase the mortality rate of MVA. Due to the limitations in separating studies, these deaths include pre-hospital and in-hospital deaths. If these deaths are preventable, interventions at all levels of prevention can reduce the mortality.

Increasing both mortality rate and injuries severity due to obesity and overweight probably means that obesity both increases the immediate mortality rate due to the severity of the injuries and the degree of complications and adverse effects in the injuries. The high severity of injuries in obese and overweight people indicates that either these people use protective equipment less frequently for any reason (lack of use, disproportion), or their physical characteristics cause severe injuries. Therefore, the results necessitate interventional measures and special prevention.

With increasing MVA in the countries, the findings of this study can be used to think out better preventive measures at all levels. By clarifying the results of this study, we can better understand the vulnerability of obese and overweight people, and help reduce the damage to these groups. The prediction of mortality and injuries and their severity in MVA with obesity, overweight or BMI variable determines the need for designing prevention programs at all levels. One of the most important programs is prevention at the first level with designing community-based training programs focusing on vulnerable people, and the need for designing newer car safety systems and promoting pre-hospital, hospital, and out-hospital care.
